# Oil Content, Fatty Acid and Phenolic Profiles of Some Olive Varieties Growing in Lebanon

**DOI:** 10.3389/fnut.2019.00094

**Published:** 2019-07-04

**Authors:** Milad El Riachy, Athar Hamade, Rabih Ayoub, Faten Dandachi, Lamis Chalak

**Affiliations:** ^1^Department of Olive and Olive Oil, Lebanese Agricultural Research Institute, Tal Amara, Lebanon; ^2^Faculty of Agronomy, The Lebanese University, Beirut, Lebanon

**Keywords:** *Olea europaea* L., variety characterization, fruit ripening, oil yield, oil quality attributes

## Abstract

Olive growing in Lebanon plays an important role at both a social and economic level. Nevertheless, the quality of olive oil produced in the country is rarely addressed. In this study, oil content, fatty acid, and phenolic profiles were studied along four different ripening stages for 11 varieties of olives, including two clones of the local variety “Baladi,” in addition to nine foreign varieties (“Ascolana Tenera,” “Bella di Cerignola,” “Itrana,” “Jabaa,” “Kalamata,” “Nabali,” “Salonenque,” “Sigoise,” and “Tanche”). Oil content was determined using the Soxhlet method and Abencor system. Fatty acid composition was determined using a GC-FID, total phenols using spectrophotometry, and the phenolic profile using HPLC-DAD. Results showed that variety, fruit ripening and their interaction have a significant effect on the overall studied oil parameters. Among the studied varieties, “Kalamata” presented the higher oil content on dry matter (OCDM = 48.24%), “Baladi 1” the highest oil content on humid matter (OCHM = 27.86%), and “Tanche” the highest oil industrial yield (OIY = 19.44%). While “Tanche” recorded the highest C18:1 (71.75%), “Ascolana Tenera” showed the highest total phenols (TP = 539 mg GAE/Kg of oil), “Salonenque” the highest oleacein (121.57 mg/Kg), and “Itrana” the highest oleocanthal contents (317.68 mg/Kg). On the other hand, oil content together with C18:2 and C18:0 increased along ripening while C18:1, total phenols and the main individual phenols decreased. Although preliminary, this study highlights the good quality of olive oil produced from both local and foreign varieties growing in Lebanon and encourages further investigations on the characterization and authentication of Lebanese olive oil.

## Introduction

Edible olives originate from areas along the eastern Mediterranean shore in what is now southern Turkey, Syria, Lebanon, and Palestine since 5,000–6,000 years ago (1, 2). Lebanon is rich in indigenous and ancient olive trees and its history of olive traditions is as old as its cultural history (3, 4). The Lebanese groves are dominated by the main traditional denomination “Baladi*.”* Other old varieties are still found in some ancient groves e.g., “Ayrouni,” “Sorani,” “Dal,” “Jlot,” and “Abou Chawkeh” across the country (5, 6). A few other varieties, introduced from neighboring Arab countries, are also found such as “Nabali” which is one of the oldest olive varieties in the Middle East. During the last few decades, grooves of foreign varieties have been planted, imported from Italy (“Frantoio,” “Leccino,” “Ascolana Tenera,” “Nocellara Del Belice”), Spain (“Manzanilla de Sevilla,” “Arbequina”), Greece (“Kalamata,” “Koroneiki”), France (“Salonenque,” “Picholine”), and Algeria (“Sigoise”) (7, 8).

The interest in olive varieties with higher oil content (OC), improved fatty acid composition, mainly high monounsaturated fatty acids (MUFAs) and a high content of phenolic compounds, has increased due to its stability and health benefits (9, 10). While OC is associated with oil quantity and olive growing profitability; the proportions of the different fatty acids and phenolic compounds are associated with oil quality. For example, a high percentage of MUFA, mainly oleic acid, is a primordial factor in determining the nutritional value of the oil as it reduces the risk of atherosclerosis (11) and protects against different kinds of cancers (12). In addition, fatty acid composition influences the stability of the oil through the contribution of polyunsaturated fatty acids (PUFAs) to oil rancidity (13). On the other hand, the amount of olive oil phenolic compounds, such as oleuropein derivatives is of primary importance when evaluating its quality, as these natural antioxidants improve oil resistance to oxidation and are responsible for its sharp bitter taste (14). The pharmacological interest of olive phenolic compounds is also well-known (15, 16).

Several agro-industrial parameters may modify the OC, the fatty acid composition and phenolic content of virgin olive oil (VOO). Indeed, previous studies showed that OC, fatty acid and phenolic profiles are a built-in genetic factor. Values between 10 and 30% of oil were reported when evaluating the different accessions of the World Olive Germplasm Bank in Cordoba-Spain (17). Moreover, in the Germplasm Banks of Catalonia and Cordoba, Tous et al. (18) and Uceda et al. (19), respectively, showed that more than 70% of the variation in the fatty acids (except for linolenic acid) and several minor components, such as phenolic compounds, bitter index (K225), and oil stability, was due to genetic effects. In addition, evaluation of new varieties obtained by breeding programs showed that genotypic variance was the main contributor to the total variance of fatty acids (20, 21) and of phenolic compounds (21, 22). In addition, fruit ripening affects oil quantity and composition as early harvested olives, especially green olives, give lower oil quantity but higher content in MUFAs and antioxidants than late harvested ones (23–25). Additionally, OC and composition may vary according to the climatic conditions of the year, the irrigation regimes and the processing systems among others (23, 25–27).

In the past few years, authenticity and characterization of olive oils have been the object of numerous studies due to the importance of the protection of consumers. Various physico-chemical determinations in association with chemometric analyses have been applied: fatty acids (28–31), fatty acids and triacylglycerols (32, 33), sterols (34), phenolic compounds (35), and aromas (36). Yet little attention has been given to the authentication and characterization of Lebanese olive oils. Chehade et al. (6, 37) described some oil traits for eight Lebanese olive varieties; however, many other varieties cultivated in Lebanon are still not comprehensively assessed. In this manuscript, we report the characterization of monovarietal olive oils for 11 varieties cultivated in northern Lebanon for their OC, fatty acid and phenolic composition along fruit ripening with the perspective of evaluating and valorizing Lebanese olive oil. Expected findings will allow the development of a set of practical recommendations providing farmers with knowledge of the best varieties to be grown and the best time for olive harvesting, to further improve Lebanese production of olive oil in terms of quantity and quality.

## Materials and Methods

### Plant Material and Extraction of Olive Oil

Olive fruits were collected from 11 varieties growing in Abdeh station of the Lebanese Agricultural Research Institute (LARI) during the 2015 olive harvest season. This station, located at 18 m a.s.l., 34°31'0“ N and 35°58'0” E, has been hosting an international olive collection of 72 olive varieties, initially collected from Lebanon, Arabic, and European countries, since the early seventies. The Abdeh region is characterized by a typical Mediterranean climate with a dry summer from June to September. The average annual precipitation sums 870 mm, the soil is a clay type soil with 25% sand, 15% silt, and 60% clay with 2% organic matter content. The olive trees in the Abdeh collection are rainfed.

The 11 genotypes considered included two clones of the local traditional variety, “Baladi 1” and “Baladi 2,” as previously differentiated by Chalak et al. (38), in addition to nine foreign varieties i.e., “Nabali” and “Jabaa” (from Palestine), “Kalamata” (derived from Greece), “Salonenque” and “Tanche” (from France), “Ascolana Tenera,” “Bella di Cerignola” and “Itrana” (originating from Italy), and “Sigoise” (derived from Algeria) (2, 39–41). For each variety, three fruit samples were collected from three trees, at different harvesting dates, based on fruit skin color (0 = deep or dark green; 1 = yellowish-green; 2 = yellowish with reddish spots; 3 = reddish or light violet; and 4 = black), and the ripening index (RI). RI was determined on samples of 100 fruits according to the method described by Uceda and Hermoso (42) and modified by El Riachy et al. (43), following the formula:

$$\begin{array}{l} \text{RI}=\frac{\left((\text{0 }\times \;{\text{n}}_{0})\text{ + }(\text{1 }\times \;{\text{n}}_{1})\text{ + }(\text{2 }\times \;{\text{n}}_{2})\text{ + }(\text{3 }\times \;{\text{n}}_{3})\text{ + }(\text{4 }\times \;{\text{n}}_{4})\right)}{\text{100}}; \end{array}$$

where, n_0_ to n_4_, is the total number of olive fruits in each category. Then, RI was categorized to the following five categories: RI ≤ 0.5 (RI0), 0.5 ≥ RI ≤ 1.5 (RI1), 1.5 ≥ RI ≤ 2 (RI2), 2.5 ≥ RI ≤ 3.5 (RI3), and finally RI ≥ 3.5 (RI4).

The olive oil was obtained using the Abencor system (MC2 Ingenierías y Sistemas, Sevilla, Spain). For each sample, ~500 g of fruits were ground to a paste using a hammer mill, stirred in the thermobeater for 30 min at 28 ± 1°C, and then centrifuged for 2 min to separate the oil, which was collected and left to decant in graduated cylinders. OIY was calculated using the following formula (44):

$$\begin{array}{l} \text{OIY}=\frac{\text{V }\times \text{ 0}.\text{915 }\times \text{ 100 }}{\text{W}}; \end{array}$$

where, V is the volume of oil obtained; and W is the weight of the processed olive paste. Finally, the oil extracted was collected and stored in glass vials in darkness at −20°C until analysis.

### Chemical Reagents

The chemical reagents used for VOO characterization were of GC or HPLC grade. Methanol, acetonitrile, *o*-phosphoric acid and the Folin-Ciocateu (F-C) reagent were provided from Sigma-Aldrich (Steinheim, Germany), *n*-hexane from Hipersolv Chromanorm (Pennsylvania, USA), Na_2_CO_3_ anhydrous from Acros organics (New Jersey, USA) and KOH from Fisher Scientific (Loughborough, UK). Regarding the commercial standards, methyl palmitate, methyl palmitoleate, methyl stearate, methyl oleate, methyl linoleate, methyl linolenate, hydroxytyrosol, tyrosol, *p*-coumaric acid, luteolin, and apigenin were purchased from Sigma-Aldrich (Steinheim, Germany) and Fluka (Steinheim, Germany), vanillic and syringic acid standards were obtained from Acros organics (New Jersey, USA), gallic acid from Fisher Scientific (Loughborough, UK), and oleacein and oleocanthal were supplied by Prof. Prokopios Magiatis (Athens, Greece). Finally, deionized water (18 MΩ cm) from a Milli-Q water purification system (Millipore, Bedford, MA, USA) was used to prepare the mobile phases for HPLC analysis.

### Determination of the Oil Content (OC) in the Olive Paste Using Soxhlet Method

OC in the olive paste was determined using a Gerhardt Soxhlet instrument (Gerhardt Soxtherm SE−416, Germany) (45). From each sample, an aliquot of 50–60 g of the olive paste was dried at 105°C to weight stability using a rapid moisture analyzer (Metler Toledo HS 153, Switzerland). One gram of the dry paste was placed on a filter paper which was folded and closed tightly using a cotton wire. Each prepared sample was placed in previously weighed soxhlet beakers containing three boiling chips. Petroleum benzene was used as a solvent. The total program length was 2 h and 20 min. When the process ended, the samples were thrown out, and the remaining solvent was eliminated by placing the beakers under a fume hood overnight, then, the beakers were dried in an oven at 105°C for 1.5 h in a desiccator for 45 min before being weighed. OC was calculated from both dry matter (OCDM) and humid matter basis (OCHM) as follows:

$$\begin{array}{l} \begin{array}{l} \text{ Oil content on dry matter }(\text{OCDM})=\frac{{\text{m}}_{2}\;-\;{\text{m}}_{1}}{\text{Sample weight}}\times \text{100}\\ \text{Oil content on humid matter }(\text{OCHM})=\frac{\left(\text{100 }-\text{ MC}\right)\;\times \text{ OCDM}}{\text{100}} \end{array} \end{array}$$

where, m_1_ = beaker weight before extraction; m_2_ = beaker weight after extraction and MC = moisture content.

### Determination of Fatty Acid Profile

Fatty acid methyl esters (FAMEs) were prepared based on a cold transmethylation reference method (46). A sample of 0.1 g oil was shaken manually with 2 mL *n-*hexane for 2 s then 0.2 mL of methanolic solution (2 N) of potassium hydroxide was added. The sample was mixed with a vortex for 1 min (1,400 rpm), before resting for 5 min. A volume of 975 μL of the upper phase that contains the FAME was transferred to 1.5 mL vials with 25 μL of external standard (nonadecanoate methyl ester 1,000 ppm). The separation of FAMEs was carried out using a Shimadzu gas chromatograph (GC-2010 Plus) equipped with a flame ionization detector (FID). A fused silica capillary column (DB-wax; Agilent Technologies, Wilmington, DE; 30 m length ×0.25 mm i.d. and 0.25 μm of film thickness) was used. Nitrogen was used as a carrier gas with a flow rate of 1.69 mL/min. As for the chromatographic gradient, the initial oven temperature was kept at 165°C for 15 min and then programmed to rise at 5°C/min up to 200°C, maintained for 2 min, and followed by a second gradient of 5°C/min to a final temperature of 240°C, which was held for 5 min. The injector and detector temperatures were 250 and 280°C, respectively. Hydrogen and compressed air were used for the flame detector. Finally, the injection volume was 1 μL with a split ratio of 50. Identification of the different fatty acids was achieved by a comparison of their retention times with those of authentic standards. Results were expressed as percentages of total fatty acids. Finally, the calculated sums and ratios were; saturated fatty acid (SFA), MUFA, PUFA, MUFA/PUFA, MUFA/SFA, PUFA/SFA, C16:0/C18:2, C18:1/C18:2, and C18:2/C18:3.

### Extraction of Phenolic Compounds

Prior analysis samples were left to thaw at room temperature. Meanwhile, an internal standard solution was prepared by dissolving 15 mg of syringic acid in 10 mL of 60:40 v/v methanol–water. Then, 1 mL from this solution was diluted in a 25 mL volumetric flask with 60:40 v/v methanol–water.

The phenolic compounds in the olive oil were extracted using a modification of the procedure described by Montedoro et al. (47). An amount of 3 g of olive oil was shaken manually with 2 mL of *n*-hexane for 15 s. Then, a volume of 1.75 mL of 60:40 (v/v) methanol–water mixture was added together with 0.25 mL of the internal standard solution and shaken for 2 min to undergo the first extraction. For the second extraction, 2 mL of methanol/water (60/40) was added and shaken for 2 min. The extracts from both extractions were combined and placed in the dark at −20°C for further determinations.

### Spectrophotometric Estimation of Total Phenols

The total phenols (TP) content of the oil extracts was determined according to the Folin-Ciocalteu spectrophotometric method (48). Briefly, 20 μL of the sample (with prior 1:50 dilution with water) was, in this order, mixed with 1.58 mL of water, 0.3 mL of 20% (w/v) Na_2_CO_3_ aqueous solution, and 0.1 mL of F–C reagent, and heated in an oven for 5 min at 50°C. Then, the resulting solution was allowed to stand for 30 min. The reaction product was spectrophotometrically monitored at 765 nm by a jenway UV/Vis spectrophotometer (Staffordshire, ST15 OSA, UK). A nine level calibration curve was prepared using gallic acid as a commercial standard (*r*^2^ = 0.998), and the results were expressed as mg gallic acid equivalent (GAE)/Kg of oil.

### Chromatographic Analysis of Phenols by HPLC-DAD

The produced extract was also used to determine the following eight individual phenolic compounds: hydroxytyrosol, tyrosol, vanillic acid, *p*-coumaric acid, oleacein, oleocanthal, luteolin, and apigenin. The extracted phenolic fraction was injected in triplicate in a Shimadzu High Performance Liquid Chromatograph (HPLC) equipped with an automatic injector, a column oven and a diode array UV detector (DAD). Separation of individual phenols was achieved on a Microsorb-MV 100 C18 column (250 × 4.6 id mm, 5 μ particle size), maintained at 40°C. The injection volume was 20 μL and the flow rate 1.0 mL/min. Mobile phases were 0.2% *o*-phosphoric acid in water (mobile phase A) and a mixture methanol-acetonitrile (50:50, v/v) (B). The initial concentrations were 96% of A and 4% of B and the gradient was changed as follows: the concentration of B was increased to 50% in 40 min, increased to 60% in 5 min, and to 100% in 15 min, and maintained for 10 min. Initial conditions were reached in 7 min. The identification of individual olive oil phenols was performed at 280 nm, on the basis of their maximum absorption and retention times compared to those of commercial standard compounds. Phenolic compounds quantification was achieved using syringic acid as an internal standard and 9 point calibration curves of authentic standards. Results were expressed as mg of the target analyte per Kg of oil.

### Statistical Analysis

The total number of samples was 129 samples. Each sample was loaded in triplicate for individual phenol analysis, in duplicate for fat content and for fatty acid determination, and just once for the TP estimation. Multivariate analysis of variance (MANOVA) was used to assess the combined effect of variety, fruit ripening, and their interaction on the different sets of variables; and one-way analysis of variance (ANOVA) was used to test the significance of the effect of the studied factors and their interaction on each parameter studied. For all traits studied, trait means, coefficient of variation, and standard error were calculated; while means were compared using Tukey's HSD test at (*p* < 0.05). A principal component analysis (PCA) was also performed in order to determine the degree of contribution of each of the characters to the total variation and to highlight the effect of the studied factors on those traits (49). Data processing was performed using the Statistics (Analytical Software, Tallahasse, FL, USA) and Unscrambler (CAMO A/S, Trodheim, Norway) statistical packages.

## Results

The combined effects of the variety, the fruit ripening and their interaction were assessed for the sets of variables related to OC, fatty acids and phenolic composition. The results of the MANOVA (Table 1) showed a highly significant effect of the interaction variety × fruit ripening (*p* < 0.001) on the three sets of variables. Similarly, each factor alone also showed a highly significant effect (*p* < 0.001) on these sets of variables. In this case, the variety and its associated error exhibited a more remarkable effect, expressed as partial η^2^, while 64% of the change in the OC set can be accounted for by the variety and its associated error; 83% in the fatty acid composition set; and 69% in the phenolic composition set. Moreover, the two studied factors and their interaction showed a very high power (=1) sufficient to detect such effects.

**Table 1:** Results of the Multivariate Analysis of Variance (MANOVA) of the three sets of variables: oil content (OC), fatty acids, and phenolics composition.

**Parameters**	**Interaction**	**Wilk's ∧**	**F**	**Partial η^2^**	**Power**
OC	Variety	0.02	14.84***	0.64	1
	Fruit ripening	0.15	18.18***	0.47	1
	Variety × fruit ripening	0.12	2.01***	0.41	1
Fatty acids	Variety	0.00	36.07***	0.83	1
	Fruit ripening	0.02	15.81***	0.74	1
	Variety × fruit ripening	0.00	4.20***	0.60	1
Phenolic compounds	Variety	0	21.17***	0.69	1
	Fruit ripening	0.14	7.85***	0.49	1
	Variety × Fruit ripening	0	5.34***	0.66	1

*** *P < 0.001*.

To have more advanced information about the effects of the variety, the fruit ripening and their interaction on each of the studied variables, the Tests of Between-Subjects Effects, which are similar to many ANOVAs, were used (Table 2). Results highlighted the relative importance, expressed as percentages of the total sum of squares, of variety (29.38–92.32%), fruit ripening (0.38–55.07%), and their interaction (1.91–39.27%); with however, a greater contribution attributed to variety except in case of C16:0/C18:2 where fruit ripening recorded the greater contribution and in case of apigenin where the interaction variety × fruit ripening showed the greater contribution. The interaction variety × fruit ripening significantly affected MC and OIY (*p* < 0.0125); C16:0, C16:1, SFA, MUFA/PUFA, MUFA/SFA, PUFA/SFA, C16:0/C18:2, and C18:1/C18:2 (*p* < 0.003); and TP and all individual phenols studied (*p* < 0.005). As per each factor alone, significant differences were observed between varieties for OC traits, fatty acids, and phenolic composition. Similarly, significant differences were observed according to the fruit ripening for OC traits, C16:1, C18:1, C18:2, MUFA, PUFA, MUFA/PUFA, PUFA/SFA, C16:0/C18:2, C18:1/C18:2, C18:2/C18:3, TP, vanillic acid, oleacein, oleocanthal, and apigenin.

**Table 2:** Relative importance of varieties and ripening index expressed as percentages of total sum of squares and significance in the ANOVA for different traits under study.

**Parameters**	**Variety**	**Fruit ripening**	**Interaction**	**Error**	**CV**	**Mean**	**SE**
MC (%)	54.03*	18.79*	11.57*	15.61	15.38	46.72	1.84
OCDM (%)	50.16*	20.33*	7.57	21.94	15.44	41.61	2.24
OCHM (%)	64.37*	9.92*	8.57	17.14	23.83	22.20	1.33
OIY (%)	51.95*	20.16*	12.94*	14.95	31.75	13.41	1.41
C16:0 (%)	77.95**	1.74	8.46**	11.85	13.83	14.98	0.49
C16:1 (%)	78.41**	3.52**	14.5**	3.57	68.46	0.97	0.08
C18:0 (%)	92.32**	0.48	1.91	5.29	36.32	3.00	0.15
C18:1 (%)	75.97**	12.7**	3.89	7.45	7.79	65.97	1.04
C18:2 (%)	58.16**	31.42**	4.02	6.40	30.22	12.80	0.69
C18:3 (%)	79.82**	0.50	6.72	12.96	19.21	0.88	0.05
SFA (%)	77.37**	1.15	10.27**	11.21	8.26	18.85	0.45
MUFA (%)	75.86**	13.2**	3.19	7.76	6.91	67.47	0.99
PUFA (%)	59.55**	29.9**	4.04	6.51	28.80	13.68	0.71
MUFA/PUFA	48.29**	38.18**	7.02**	6.51	33.20	5.46	0.32
MUFA/SFA	83.38**	0.38	7**	9.24	18.67	3.68	0.14
PUFA/SFA	48.41**	37.48**	7.51**	6.61	31.03	0.74	0.04
C16:0/C18:2	29.38**	55.07**	8.39**	7.16	28.44	1.27	0.07
C18:1/C18:2	46.75**	39.38**	7.56**	6.31	35.95	5.78	0.36
C18:2/C18:3	49.16**	39.77**	4.03	7.04	28.93	14.90	0.76
TP (mg GAE/Kg of oil)	46.95***	9.81***	31.77***	11.46	42.81	363	37.55
Hydroxytyrosol (mg/Kg)	39.8***	2.21	25.69***	32.31	102.26	6.80	1.00
Tyrosol (mg/Kg)	56.26***	1.25	27.7***	14.78	82.24	3.75	0.74
Vanillic acid (mg/Kg)	88.18***	1.27***	5.81***	4.73	36.98	3.20	0.24
*P*-coumaric acid (mg/Kg)	39.09***	0.21	30.68***	30.01	136.72	2.73	0.26
Oleacein (mg/Kg)	55.4***	1.86***	34.37***	8.36	76.14	46.20	8.99
Oleocanthal (mg/Kg)	65.26***	5.07***	25.19***	4.48	69.75	132.66	13.68
Luteolin (mg/Kg)	37.39***	4.18	33.12***	25.31	52.77	4.33	0.86
Apigenin (mg/Kg)	34.1***	4.31***	39.27***	22.31	66.05	6.89	1.00

MC, moisture content; OCDM, oil content on dry matter; OCHM, oil content on humid matter; OIY, oil industrial yield; C16:0, palmitic; C16:1, palmitoleic; C18:0, stearic; C18:1, oleic; C18:2, linoleic; C18:3, linolenic; SFA, saturated fatty acid; MUFA, monounsaturated fatty acid; PUFA, polyunsaturated fatty acid; TP, total phenols; GAE, Gallic acid equivalent; RI, ripening index;

* p < 0.0125;

** p < 0.003;

*** *p < 0,005 (Considering the Bonferroni corrections)*.

Mean comparisons (Table 3) showed that “Kalamata” recorded the highest OCDM (48.24%), “Baladi 1” the highest OCHM (27.86%), and “Tanche” the highest OIY (19.44%). As per the fatty acid composition, “Tanche” was characterized by the highest percentage of MUFA (72.43%) as it recorded the highest C18:1 (71.75%), the most important fatty acid characteristic of olive oil. On the other hand, “Jabaa” was characterized by higher percentages of PUFA (20.59%) due to the high percentages of C18:2 (19.47%) and C18:3 (1.12%) recorded in this variety. As per the SFA, “Saloneque” presented the highest percentage of C16:0 (17.34%) and “Bella di Cerignola” the highest C18:0 (4.35%), while the highest SFA was recorded in “Saloneque” (20.48%). Regarding the different ratios that strongly influence olive oil oxidative stability and health benefits, “Tanche” presented the highest MUFA/PUFA (7.02), the highest C18:1/C18:2 (7.64) and together with “Sigoise” the highest MUFA/SFA (4.62 and 4.74, respectively); and “Nabali” recorded the lowest PUFA/SFA (0.64). Regarding phenolic compounds, “Ascolana Tenera” presented the highest content of TP (539 mg/Kg), hydroxytyrosol (9.1 mg/Kg), tyrosol (8.97 mg/Kg), and vanillic acid (3.46 mg/Kg); “Itrana” the highest values of *p*-coumaric acid (2.59 mg/Kg) and oleocanthal (317.68 mg/Kg); “Saloneque” the highest content of oleacein (121.57 mg/Kg); and “Jabaa” the richest in Apigenin (7.93 mg/Kg).

**Table 3:** OC, fatty acids and phenolic compounds traits variability among the studied varieties cultivated in Lebanon.

**Variables**	**“Ascolana Tenera”**	**“Baladi 1”**	**“Baladi 2”**	**“Bella di Cerignola”**	**“Itrana”**	**“Jabaa”**	**“Kalamata”**	**“Nabali”**	**“Saloneque”**	**“Sigoise”**	**“Tanche”**	**IOC limits**
MC (%)	53.22^a^	38.92^f^	42.15^ef^	47.04^cd^	43.60^de^	54.77^a^	51.59^ab^	48.39^bc^	47.41^bcd^	44.93^cde^	42.55^ef^	–
OCDM (%)	38.09^cd^	45.72^a^	44.57^ab^	38.56^cd^	37.19^d^	31.42^e^	48.24^a^	43.4^abc^	45.67^a^	40.02^bcd^	44.05^ab^	–
OCHM (%)	17.78^f^	27.86^a^	25.56^ab^	20.38^ef^	20.91^def^	14.18^g^	23.29^bcde^	22.23^cde^	23.89^bcd^	22.11^de^	25.36^abc^	–
OIY (%)	11.06^cd^	18.19^a^	17.17^ab^	11.40^cd^	14.47^bc^	8.37^de^	14.47^bc^	9.57^de^	14.18^bc^	7.14^d^	19.44^a^	–
C16:0 (%)	17.08^a^	13.88^b^	14.03^b^	12.89^b^	16.59^a^	17.29^a^	13.19^b^	16.25^a^	17.34^a^	12.87^b^	13.5^b^	7.5–20
C16:1 (%)	2.04^a^	0.47^g^	0.47^g^	0.34^g^	1.32^c^	1.64^b^	0.76^ef^	0.96^d^	1.19^c^	0.94^de^	0.68^f^	0.3–3.5
C18:0 (%)	1.97^d^	4.05^a^	4.17^a^	4.35^a^	2.19^d^	1.88^d^	2.25^d^	2.74^c^	3.14^b^	2.16^d^	2.21^d^	0.5–5
C18:1 (%)	63.94^d^	67.63^bc^	67.07^c^	70.14^ab^	66.49^c^	57.14^e^	68.08^bc^	66.98^c^	57.59^e^	70.08^ab^	71.75^a^	55–83
C18:2 (%)	12.5^bc^	11.77^cd^	12.03^cd^	10.70^cd^	11.54^cde^	19.47^a^	13.75^b^	11.36^cde^	17.94^a^	11.99^cd^	9.96^e^	3.5–21
C18:3 (%)	1.02^ab^	0.64^d^	0.64^d^	0.94^bc^	0.83^c^	1.12^a^	0.83^c^	0.70^d^	1.10^a^	1.08^a^	0.9^c^	<1
SFA (%)	19.05^b^	17.93^cd^	18.21^bcd^	17.24^d^	18.78^bc^	19.17^b^	15.44^e^	19^bc^	20.48^a^	15.03^e^	15.71^e^	–
MUFA (%)	65.98^e^	68.10^cde^	67.54^de^	70.48^abc^	67.8^de^	58.78^f^	68.84^bcd^	67.94^de^	58.79^f^	71.02^ab^	72.43^a^	–
PUFA (%)	13.52^bc^	12.41^cde^	12.67^cd^	11.64^de^	12.37^cde^	20.59^a^	14.58^b^	12.07^cde^	19.03^a^	13.08^bcd^	10.85^e^	–
MUFA/PUFA	5.07^cd^	5.8^bc^	5.65^cd^	6.54^ab^	5.7^cd^	2.96^e^	4.99^d^	5.76^cd^	3.17^e^	5.71^cd^	7.02^a^	–
MUFA/SFA	3.47^c^	3.63^c^	3.57^c^	4.11^b^	3.61^c^	3.07^e^	4.5^a^	3.59^c^	2.88^e^	4.74^a^	4.62^a^	–
PUFA/SFA	0.71^c^	0.66^c^	0.66^c^	0.67^c^	0.66^c^	1.07^a^	0.96^b^	0.64^c^	0.93^b^	0.89^b^	0.7^c^	–
C16:0/C18:2	1.42^ab^	1.24^c^	1.21^c^	1.29^bc^	1.49^a^	0.91^e^	1.02^de^	1.46^a^	0.98^e^	1.17^cd^	1.44^ab^	–
C18:1/C18:2	5.34^c^	6.06^bc^	5.88^bc^	7.15^a^	6.04^bc^	3.06^d^	5.26^c^	6.04^bc^	3.3^d^	6.23^b^	7.64^a^	–
C18:2/C18:3	12.28^cd^	18.38^a^	19.05^a^	11.4^d^	14.02^c^	17.47^ab^	16.5^b^	16.19^b^	16.34^b^	11.31^d^	11.07^d^	–
TP (mg GAE/Kg of oil)	539^a^	321^cd^	301^cd^	207^ef^	469^ab^	384^bc^	285^de^	176^f^	157^f^	275^de^	276^de^	–
Hydroxytyrosol (mg/Kg)	9.1^a^	4.3^bcd^	3.69^bcd^	2.95^d^	4.86^bcd^	5.92^b^	3.09^cd^	5.51^bc^	3.28^cd^	3.31^cd^	4.92^bcd^	
Tyrosol (mg/Kg)	8.97^a^	3.25^c^	2.19^cd^	0.98^d^	6.04^b^	1.89^cd^	3.04^c^	2.31^**cd**^	2.36^cd^	3.12^c^	2.09^cd^	–
Vanillic acid (mg/Kg)	3.46^a^	2.8^bc^	2.72^cd^	2.87^bc^	2.88^bc^	3.34^ab^	2.4^cd^	2.16^d^	2.21^d^	2.36^cd^	2.72^cd^	
*p*-Coumaric acid (mg/Kg)	1.74^bcd^	2.22^ab^	1.95^abc^	1.58^cd^	2.59^a^	1.38^cd^	1.69^bcd^	1.62^bcd^	1.47^cd^	1.15^d^	2.42^a^	–
Oleacein (mg/Kg)	51.41^bc^	26.97^d^	27.78^d^	49.62^bc^	108.03^a^	14.3^d^	32.54^cd^	58.2^b^	121.57^a^	62.75^b^	62.49^b^	–
Oleocanthal (mg/Kg)	109.89^cd^	73.53^efgh^	48.36^h^	132.81^bc^	317.68^a^	102.98^cde^	156.24^b^	90.03^def^	87.43^defg^	55.26^gh^	59.21^fgh^	–
Luteolin (mg/Kg)	2.98^def^	7.19^a^	3.94^bcdef^	2.65^ef^	4.4^bcde^	5.35^abc^	4.87^bcd^	3.33^cdef^	1.96^f^	5.81^ab^	3.02^def^	–
Apigenin (mg/Kg)	4.71^bcde^	6.82^ab^	4.18^cde^	3.7^cde^	7.89^a^	7.93^a^	2.79^e^	5.03^bcde^	3.12^de^	5.25^bcd^	6.08^abc^	–

*IOC, international olive council; MC, moisture content; OCDM, oil content on dry matter; OCHM, oil content on humid matter; OIY, oil industrial yield; C16:0, palmitic acid; C16:1, palmitoleic acid; C18:0, stearic acid; C18:1, oleic acid; C18:2, linoleic acid; C18:3, linolenic acid; SFA, saturated fatty acid; MUFA, monounsaturated fatty acid; PUFA, polyunsaturated fatty acid; TP, total phenols; GAE, gallic acid equivalent. Different letters in a row indicate significant differences (Tukey's HSD test)*.

OC traits and the majority of fatty acids and phenolic compounds appeared to be significantly affected by the fruit ripening (Table 4). OC assessed as OCDM, OCHM, and OIY, showed a general increase along the ripening process; although, OCHM and OIY decreased with the increase of moisture content after rainfall. Indeed, OCDM increased progressively from 37.27% at RI = 1 to 45.75 at RI = 4; however, OCHM and OIY, increased from RI = 1 (19.88 and 9.51%, respectively) to RI = 3 (23.42 and 15.30%, respectively) before decreasing at RI = 4 (22.52 and 13.96%, respectively). Among fatty acids, C16:0 and C18:1 decreased; although the difference was only significant in case of C18:1 (*p* < 0.003). In contrast, C16:1, C18:0, and C18:2 increased with a significant difference only in the case of C16:1 and C18:2. Similarly, the studied phenolic compounds displayed different tendencies along ripening too. TP, oleacein and oleocanthal significantly decreased from 387 mg GAE/Kg, 61.87 mg/Kg, and 136.28 mg/Kg, respectively at RI = 1 to 250 mg GAE/Kg, 46.57 mg/Kg, and 81.13 mg/Kg, respectively at RI = 4. The rest of the phenols increased until RI = 2 in case of hydroxytyrosol, *p*-coumaric acid, luteolin, and apigenin and until RI = 3 in case of tyrosol and vanillic acid, before decreasing at RI = 4; although the difference was only significant (*p* < 0.005) in the case of vanillic acid and apigenin.

**Table 4:** Evolution of OC, fatty acids and phenolic compounds traits along ripening.

	**RI**			
**Variables**	**1**	**2**	**3**	**4**
MC (%)	44.15^b^	43.65^c^	45.61^bc^	50.81^a^
OCDM (%)	37.27^c^	40.98^b^	43.01^b^	45.75^a^
OCHM (%)	19.88^b^	23.10^a^	23.42^a^	22.52^a^
OIY (%)	9.51^b^	15.11^a^	15.30^a^	13.96^a^
C16:0 (%)	15.41^a^	15.22^ab^	14.78^bc^	14.5^c^
C16:1 (%)	0.87^b^	0.92^b^	1.02^a^	1.06^a^
C18:0 (%)	2.73^b^	2.90^ab^	2.88^ab^	2.94^a^
C18:1 (%)	68.92^a^	66.03^b^	64.46^c^	64.41^c^
C18:2 (%)	9.79^c^	12.75^b^	14.65^a^	15.38^a^
C18:3 (%)	0.91^a^	0.86^a^	0.88^a^	0.90^a^
SFA (%)	18.13^a^	18.12^a^	17.66^ab^	17.45^b^
MUFA (%)	69.79^a^	66.95^b^	65.48^c^	65.46^c^
PUFA (%)	10.69^c^	13.60^b^	15.53^a^	16.29^a^
MUFA/PUFA	7.00^a^	5.27^b^	4.44^c^	4.31^c^
MUFA/SFA	3.85^a^	3.72^a^	3.76^a^	3.82^a^
PUFA/SFA	0.58^d^	0.74^c^	0.88^b^	0.93^a^
C16:0/C18:2	1.65^a^	1.24^b^	1.04^c^	0.97^c^
C18:1/C18:2	7.61^a^	5.57^b^	4.65^c^	4.49^c^
C18:2/C18:3	11.02^c^	15.07^b^	16.93^a^	17.45^a^
TP (mg GAE/Kg of oil)	387^a^	305^b^	268^bc^	250^c^
Hydroxytyrosol (mg/Kg)	4.21^ab^	5.16^a^	4.93^ab^	3.84^b^
Tyrosol (mg/Kg)	2.8^a^	3.5^a^	3.53^a^	2.82^a^
Vanillic acid (mg/Kg)	2.67^a^	2.79^a^	2.93^a^	2.37^b^
*p*-Coumaric acid (mg/Kg)	1.82^a^	1.91^a^	1.79^a^	1.74^a^
Oleacein (mg/Kg)	61.87^a^	60.95^a^	54.12^ab^	46.57^b^
Oleocanthal (mg/Kg)	136.28^a^	130.72^a^	104.59^b^	81.13^c^
Luteolin (mg/Kg)	4.48^ab^	4.56^a^	3.81^ab^	3.53^b^
Apigenin (mg/Kg)	5.49^ab^	6.07^a^	4.84^b^	4.47^b^

*RI, ripening index; MC, moisture content; OCDM, oil content on dry matter; OCHM, oil content on humid matter; OIY, oil industrial yield; C16:0, palmitic acid; C16:1, palmitoleic acid; C18:0, stearic acid; C18:1, oleic acid; C18:2, linoleic acid; C18:3, linolenic acid; SFA, saturated fatty acid; MUFA, monounsaturated fatty acid; PUFA, polyunsaturated fatty acid; TP, total phenols; GAE, gallic acid equivalent. Different letters in a row indicate significant differences (Tukey's HSD test)*.

**Figure 1:**
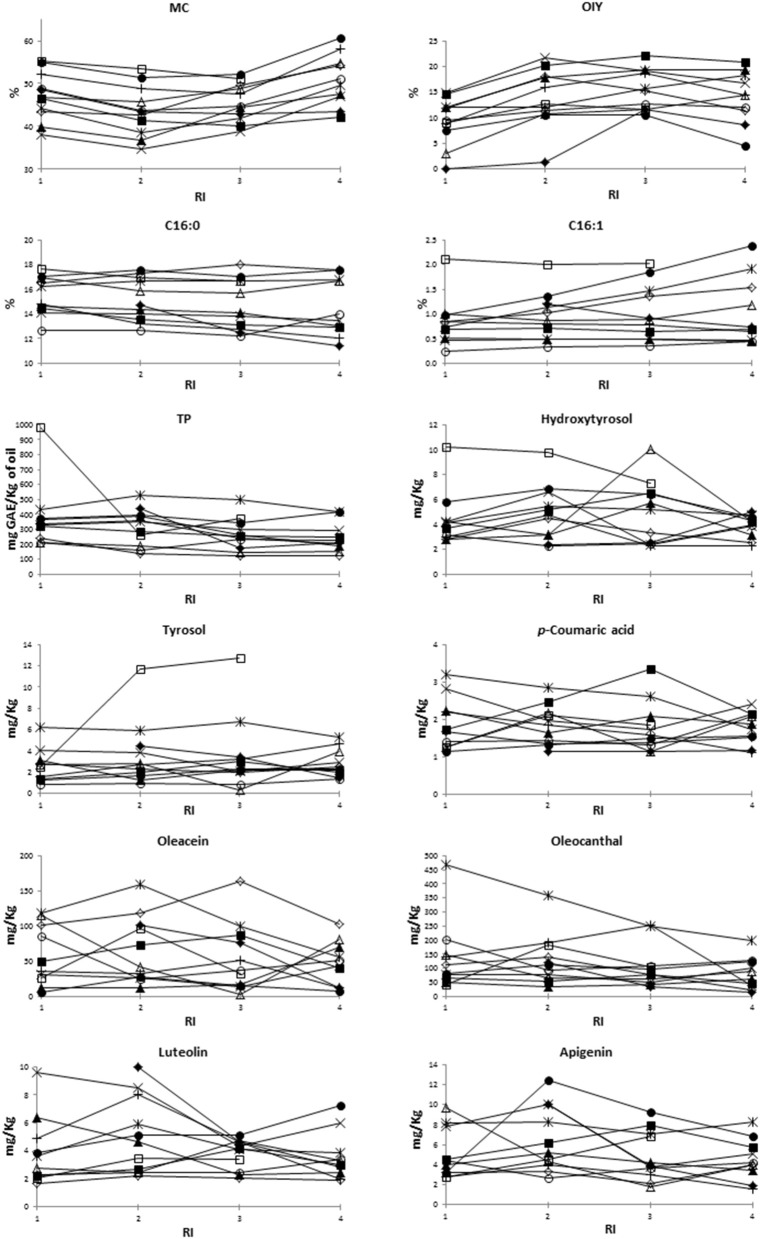
Evolution pattern along ripening of the different studied traits. MC, moisture content; OIY, oil industrial yield; TP, total phenols; RI, ripening index. □, “Ascolana Tenera;” ×, “Baladi 1;” ▴, “Baladi 2;” ◦, “Bella di Cerignola;” *, “Itrana;” •, “Jabaa;” +, “Kalamata;” ▵, “Nabali;” ♢”, “Salonenque;” ♦, “Sigoise;” ■, “Tanche”.

The general evolution pattern described above along ripening was in some studied traits different between varieties (Figure 1). For instance, OIY increased along ripening in “Baladi 2,” “Itrana,” and “Nabali”; however, it increased until RI = 2 in case of “Ascolana,” “Baladi 1,” “Jabaa,” and Salonenque” and until RI = 3 in “Bella di Cerignola,” “Tanche,” “Kalamata,” and “Sigoise” before decreasing later on. Among fatty acids, C16:0 decreased along ripening in “Ascolana,” “Baladi 1,” “Baladi 2,” “Kalamata,” “Sigoise,” and “Tanche”; decreased until RI = 3 and then increased in “Bella di Cerignola” and “Nabali”; but increased until RI = 3 and then decreased slightly in “Itrana,” and “Salonenque.” While TP generally decreased along ripening in almost all varieties; it increased in “Bella di Cerignola” and Jabaa. The evolution of TP was noticeable in “Baladi 1,” “Baladi 2,” “Itrana,” and “Kalamata” as it increased from RI = 1 to RI = 2 and then decreased to values lower than those recorded at RI = 1. As per the most concentrated individual phenols, oleacein decreased in “Sigoise”; decreased in “Bella di Cerignola” till RI = 2 and then increased; decreased in “Baladi 1” and “Nabali” till RI = 3 and then increased; and decreased till RI = 2, increased till RI = 3 and then decreased in “Kalamata.” In contrast, oleacein content increased in “Baladi 2”; increased till RI = 2 in “Ascolana Tenera,” “Itrana,” and “Jabaa” and then decreased; and increased till RI = 3 and then decreased in “Salonenque” and “Tanche.” Oleocanthal showed the same evolution pattern as oleacein in the case of “Ascolana Tenera,” “Baladi 2,” “Bella di cerigonala,” “Itrana,” “Nabali,” and “Sigoise.” However, it increased till RI = 2, decreased at RI = 3 and increased beyond in “Baladi 1” and “Jabaa”; increased till RI = 2 and then decreased in “Salonenque”; increased till RI = 3 and then decreased in “Kalamata”; and decreased till RI = 2, increased at RI = 3 and then decreased beyond in “Tanche.”

**Figure 2:**
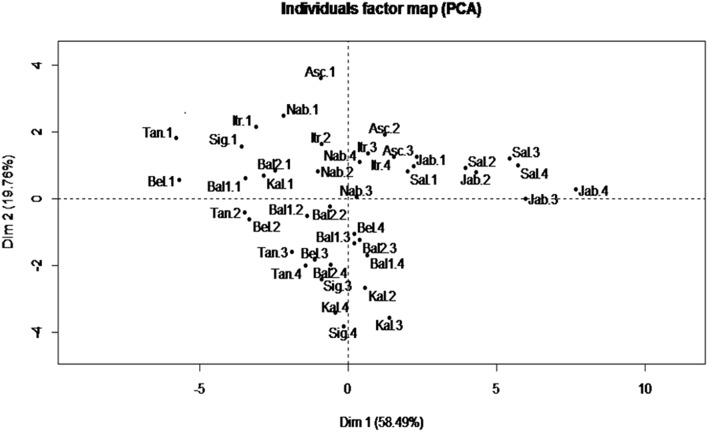
Biplot of principal components 1 and 2 based on fatty acid profile components recorded for each variety at different ripening indexes. Asc, “Ascolana Tenera;” Bal1, “Baladi 1;” Bal2, “Baladi 2;” Bel, “Bella di Cerignola;” Itr, “Itrana;” Jab, “Jabaa;” Kal, “Kalamata;” Nab, “Nabali;” Sal, “Salonenque;” Sig, “Sigoise;” Tan, “Tanche.” 1, 2, 3, and 4 are the different harvesting times.

A Principal component analysis (PCA) was performed for fatty acid traits which are recognized as the most stable indicators for characterizing a given VOO (39). The results showed that the first two PCs explained 78.25% of the total variance (Figure 2). PC1 accounted for 58.49% of the total variance with a high positive correlation with PUFA and C18:2; and a high negative correlation with MUFA and C18:1. However, PC2 accounted for 19.76%, with a high positive correlation with SFA and C16:0. In addition, in the correlation circle, a negative correlation was observed between C18:1 and MUFA from one side and C18:2 and PUFA from the other side (data not shown).

The PCA was able to differentiate more between harvesting times rather than between varieties, as it can be seen that in the individual score plot (Figure 2) the samples from harvest 1, with higher content of C16:0 and C18:1 in comparison with other harvests, are all grouped in the negative side of the PC1 and in the positive side of PC2 except the samples of “Jabaa” and “Salonenque” as these varieties are characterized by the lowest content of C18:1 (57.14 and 57.59%, respectively) and the highest content of C18:2 (19.47 and 17.94%, respectively) among all varieties studied.

## Discussion

In this study, the characterization of 11 olive varieties cultivated in Lebanon was reported at different ripening stages using 28 traits relevant to OC, fatty acid, and phenolic profile. Results showed the strong effects of variety, fruit ripening and their interaction on the majority of the studied traits. This strong genetic variability revealed here is in parallel with the results previously reported for advanced selections of the olive breeding program in Cordoba (Spain) (43, 50). Moreover, these results confirm the physical and chemical modifications occurring during fruit ripening, affecting OC, fatty acid and phenolic composition and consequently oil quality and oxidative stability (24, 51).

The OC traits, mainly OCDM (31.42–48.24%), OCHM (14.18–27.86%), and OIY (7.14–19.44%), showed high variability among varieties. Similar wide ranges of variation were observed by Chehade et al. (37) while characterizing the oil content and composition of five main Lebanese olive cultivars (“Aayrouni,” “Abou Chawkeh,” “Baladi,” “Del,” and “Soury”). These authors reported an OCHM between 17.8 and 36.4% and an OCDM between 42.5 and 56.2% in olives at spotted stage of ripening, with comparable values recorded for “Baladi” (28 ± 1% and 42.5 ± 1.8%, respectively). OCDM values increased along ripening reaching a significantly higher value at RI = 4; however, OCHM and OIY reached the maximum values at RI = 3 before decreasing later on probably due to rainfall in this period. This pattern of evolution of OC traits along ripening was also similar to the one described in other olive varieties in Spain (23, 24) and Tunisia (25, 52).

Regarding fatty acid profile, all percentages of fatty acids obtained in the present study fit, more or less, with the requirements established by the IOC for VOO, except for C18:3 which slightly exceeded the limit of IOC (*C*18:3 < 1%) in case of “Ascolana Tenera” (1.02%), “Jabaa” (1.12%), “Salonenque” (1.10%), and “Sigoise” (1.08%). Similarly, previous work found that the amounts of some fatty acids could be outside the ranges listed by IOC such as in French (53), Tunisian (54, 55), Moroccan (56), Argentinian (57), New Zealandian (58), and Italian olive varieties (59). On the other hand, our study revealed a wide variability in the percentages of the main fatty acids of olive oil in parallel with the findings of León et al. (20), Boskou (9), Diraman et al. (60), Sánchez de Medina et al. (50), and Chehade et al. (37). Oleic acid is well-recognized to be the most important fatty acid in olive oil, associated with its high nutritional value and oxidative stability (61, 62). An olive variety is considered as having a high content of oleic acid if C18:1 is about 65% and above (39). In the present study, eight of the 11 studied varieties including the local “Baladi” ones could be categorized as having high oleic acid content. In addition, “Baladi 1” and “Baladi 2” presented the lowest percentages of C18:3 (0.64%), and medium percentages of C18:2 (11.77 and 12.03%, respectively) which also increase their oxidative stability. These results are partially in agreement with those obtained by Chehade et al. (37) on the same variety (65.95% of C18:1 in 2011 but 62.44% in 2010; and 11.30% of C18:2 in 2011 but 16.39% in 2010). In addition, the results are close to those described by El Riachy et al. (63) who reported values of C18:1 between 69.42 and 71.46%, of C18:2 between 10.37 and 11.34%, and of C18:3 between 0.59 and 0.63%. These discrepancies are possibly due to the fact that the latter authors collected the samples from different altitudes (200–1,050 m a.s.l.), while, in the present study the samples were collected only from one site at a low altitude (18 m a.s.l.); and it is well known that C18:1 increases with altitude (63, 64). Regarding foreign varieties, “Sigoise” showed higher percentages of C18:1 and C18:2 in Lebanon (70.08 and 11.99%, respectively) than in Tunisia (around 68.5 and 10.02%, respectively) (65). However, “Salonenque” in Lebanon recorded higher percentages of C16:0 (17.34%) and C18:2 (17.94%) but a lower value for C18:1 (57.59%) in comparison to those recorded by Ollivier et al. (39) in France (14.55, 12.59, and 64.76%, respectively). This data variation could be attributed to the geographical area of growing and its climatic conditions which affect olive oil fatty acid profile (24).

Besides the individual fatty acids, several sums and ratios were evaluated in this study because of their importance in olive oil quality such as MUFA (61, 66). For instance, “Tanche,” “Sigoise,” and “Bella di Cerignola” presented the highest MUFA values. The local varieties “Baladi 1” and “Baladi 2” recorded lower values (68.1 and 67.54%, respectively) including values lower than those reported by Merchak et al. (64) who also collected the samples from different altitudes (0 to more than 700 m a.s.l.). The high ratios of MUFA/PUFA and C18:1/C18:2 recorded in “Tanche” and MUFA/SFA recorded in “Sigoise”; and the low PUFA/SFA ratio registered in “Nabali” are linked to the high oxidative stability and low rancidity of olive oil (13), and affects, in combination with other minor compounds, the organoleptic and health properties of VOO (9, 67).

Concerning the evolution of the different fatty acids along ripening, the results of this study showed a general decrease of C16:0 and C18:1 together with a significant increase of C18:2; in concordance with several previous studies (24, 64, 68). According to Gutiérrez et al. (23), the C16:0 level fell during the ripening process, possibly as a result of a dilution effect. However, the increase in C18:2 content could be linked to the activity of the enzyme oleate desaturase, transforming oleic acid into linoleic acid (23, 69). These changes will produce a decrease of the ratio C18:1/C18:2 together with an increase of the ratio PUFA/SFA which will have a detrimental effect on the oil oxidative stability reducing the shelf-life of olive oils obtained from late harvest.

Results of total and individual phenols also expressed a large variability among the varieties studied. These results are in agreement with previous works indicating that genetic variability is the main factor affecting the phenolic composition (22, 70). The TP values obtained for “Baladi 1” and “Baladi 2” are higher than those reported by Chehade et al. (37) and El Riachy et al. (63) for the same variety (167–249 mg GAE/kg of oil and 194–236 mg GAE/kg of oil, respectively). It is worth pointing out here the low content of secoiridoids in the local varieties in comparison to the foreign ones, which highlights the need for breeding to improve the traditional local varieties as these phenolic compounds have a detrimental effect on olive oil quality and nutraceutical properties and are very useful as selection criteria in breeding programs. Additionally, several works indicate a significant decrease of TP content during ripening (43, 54, 71) similar to the pattern described in the present paper. This decrease was most likely correlated with the increased activity of hydrolytic enzymes observed during ripening (72). In parallel, oleacein and oleocanthal also decreased from 61.87 to 46.57 mg/Kg and from 136.28 to 81.13 mg/Kg, respectively. It is worth noting that, the other studied individual phenols recorded the maximum values at RI between 2 and 3 before decreasing later on. Therefore, early harvesting (before black pigmentation, RI = 4) should be recommended to obtain higher content of phenolic compounds in addition to higher content of C18:1. These results are in partial agreement with other studies that reported an increase in TP content to a maximum level at the “spotted” and “purple” pigmentation, the content decreasing drastically as ripening progressed (24, 52).

Finally, PCA analysis showed that PC1 and PC2 explained the highest total variance (78%) when using the set of 15 traits related to fatty acid composition alone. This PCA allowed the classification of the samples according to the ripening stage with samples at RI = 1, having higher percentages of C16:0, C18:1, and MUFA, clustering together in the negative side of PC1 and positive side of PC2 (Figure 2). These results are in line with those obtained by León et al. (73) who separated 18 varieties growing in the World Olive Germplasm Bank (WOGB) of IFAPA (Cordoba, Spain) into four groups according to their fatty acid composition and to the country of origin, where the percentages of C18:1, C18:2, and saturated fatty acids were the main contributors of variation.

In conclusion, the present study reports the characterization of 11 local and foreign varieties growing under Lebanon climatic conditions. In comparison to foreign varieties, the local “Baladi 1” showed outstanding OCHM; however, both “Baladi 1” and “Baladi 2” recorded similar fatty acid composition but low phenolic composition. Indeed, many of the studied foreign varieties presented several outstanding characteristics such as “Kalamata” in terms of higher OCHM; “Tanche” in terms of higher OIY; “Tanche” and “Sigoise” in terms of higher C18:1, higher MUFA, higher MUFA/PUFA ratio, higher C18:1/C18:2, higher MUFA/SFA, and higher C18:1/C18:2; “Ascolana Tenera” and “Itrana” in terms of higher total phenols content; “Itrana” in terms of oleocanthal; and “Salonenque” in terms of oleacein. Taking into consideration that the appropriate time for harvesting is when a good balance between oil quality and oil quantity is achieved, and based on the fact that OCHM and OIY increased significantly until RI = 2 while C18:1, MUFA, MUFA/PUFA, C16:0/C18:2, C18:1/C18:2, TP, oleacein, and oleocanthal decreased along ripening, we can conclude that the best time for harvesting is at RI = 2 (when the color is yellowish green with reddish spots). This is true for all studied varieties, except “Sigoise” that must be harvested later at RI = 3 for better oil extractability. These findings on monovarietal olive oils should be confirmed over years in order to evaluate and valorize the local olive germplasm compared to the foreign one, and for the characterization and authentication of Lebanese olive oil. This is also critical in identifying the main default of the local varieties in order to start their genetic improvement through breeding programs.

## Data Availability

The raw data supporting the conclusions of this manuscript will be made available by the authors, without undue reservation, to any qualified researcher.

## Author Contributions

MR and LC contributed to the conception and design of the experiment, statistical analysis, and paper preparation. AH and RA performed sample collection and all chemical analyses. AH also contributed to the statistical analysis and data elaboration. FD participated in sample collection and experiment design.

### Conflict of Interest Statement

The authors declare that the research was conducted in the absence of any commercial or financial relationships that could be construed as a potential conflict of interest.

## Abbreviations

GAE: Gallic acid equivalent
MC: Moisture content
OC: Oil content
OCDM: Oil content on dry matter
OCHM: Oil content on humid matter
OIY: Oil industrial yield
PCA: Principal component analysis
RI: Ripening index
TP: Total phenols.
